# Rac1, caveolin-1 and vascular endothelial growth factor -mediated liver sinusoidal endothelial cell angiogenesis

**DOI:** 10.1111/j.1478-3231.2008.01898.x

**Published:** 2009-02

**Authors:** Filip Braet

**Affiliations:** Australian Key Centre for Microscopy and Microanalysis (AKCMM), The University of SydneyNSW, Australia

## Rac1 and cell morphogenesis

In this issue of *Liver International*, Yokomori *et al*. (1) detailed how Rac1, a member of the Rho-GTPases protein family, plays a key role in the migration of liver sinusoidal endothelial cells (LSECs) and in the subsequent process of capillary-like tubular formation (i.e. endothelial cell differentiation). The family of Rho-GTPases, thanks to their capacity to regulate actin cytoskeleton dynamics, generally control a wide range of cellular processes, such as cell proliferation, differentiation, motility, membrane transport and cell permeability (2). In fibroblasts and microvascular endothelial cells, Cdc42 produces microspikes and filopodia, Rac1 induces ruffles and lamellipodia, and RhoA promotes stress-fibre formation. The same research group demonstrated the role of RhoA in LSECs in controlling the fenestral diameter (3) – the fenestrae are open, membrane-bound pores surrounded by a cytoskeletal ring that control the extensive exchange between the liver sinusoidal blood and the hepatocytes. In the present study, Yokomori *et al*. show in cultured LSECs that Rac1 activity markedly increases with time after continuing exposure to vascular endothelial growth factor (VEGF). In addition, the authors present the first molecular and structural evidence that Rac1 mediates the formation of capillary-like tubular structures when LSECs were exposed to VEGF and grown on matrigel as the cell-culture substrate. The authors postulated that this peculiar cellular rearrangement of LSECs is the first morphological sign of LSEC differentiation, and as such the structural precursor in the complex cascade of LSEC angiogenesis (*vide infra*). Hence, reviewing Yokomori's data in light of the recent Rho-GTPases (Rho, Rac and Cdc42) angiogenesis findings by others [for a review, see (4)], illustrates not only the novelty of the data but also highlight that LSEC-associated Rho-GTPases might serve as potential molecular targets for treating diseases in which the liver sinusoidal microvasculature plays some major role. For example, the function of Rho-GTPases, or their related downstream effector molecules, are involved in the processes of primary or secondary liver cancer, liver cirrhosis and hepatitis (5). These are all important and severe clinical conditions in which the liver sinusoidal microcirculation is central. Potential therapeutic approaches – involving switching on or off a GTPases-mediated signal-transduction chain – have been recently discussed, reviewed and published in other relevant pathological settings such as cancer (6) and neuro-related diseases (7).

## Caveolin-1 and angiogenesis

Interestingly, the authors also observe a concurrent time-dependent increase in caveolin-1 (Cav-1) levels in their experimental setup, indicating that this membrane protein might also be an essential molecular modulator in the process of LSEC capillary-tube formation. Indeed, although all types of caveolins (Cav-1, Cav-2 and Cav-3) are similar in structure and associate with cholesterol and sphingolipids in certain areas of the cell membrane, leading to the formation of caveolae, Cav-1 is most prominently expressed in endothelial cells and fibrosblasts (8). The recent data of Grande-Garciá and Del Pozo (9) directly support the Cav-1 findings of Yokomori *et al*., as the Cav-1 and Rho GTP-ases proteins (*vide supra*) are closely involved in cell polarization and directional migration of fibroblasts (10). In view of this, it is reasonable to suggest that the same cascade of molecular networks will occur in the liver sinusoidal endothelium. It is also noteworthy that LSEC-associated Cav-1 facilitates liver sinusoidal transendothelial transport (11) and promotes in one or another way the onset of fenestrae (12). Yokomori and colleagues postulate that LSEC-associated Cav-1 has another pivotal function as a regulator of LSEC proliferation, a necessary step in the process of angiogenesis. Taking all the functions of LSEC Cav-1 together, it is clear that complex Cav-1 cross-talk signal pathways must be present within the liver sinusoidal endothelial lining. Indeed, cell-type-specific complex Cav-1 regulatory pathways exist that regulate the multiple functions of this membrane-associated protein [for a review, see (13)]. Currently, however, it remains to be seen how LSECs manage to control the balance between Cav-1-mediated transport and Cav-1-mediated cell migration and/or proliferation, or whether both occur in harmony.

## Caveolin-1, Rho family GTPases and vascular endothelial growth factor-induced angiogenesis

Migration of endothelial cells, induced by VEGF, is a critical step in angiogenesis and the Rac GTPase is known to be the critical molecular intersection during stimulation of endothelial-cell motility (4, 14). On the other hand, VEGF is a well-known, potent inducer of caveolae in microvascular endothelium (12, 15). Moreover, it has been shown that Cav-1 expression enhances formation of endothelial capillary tubules in microvascular endothelial cells (16), and manipulating Cav-1 expression, or negatively interfering with caveolae formation, adversely affects cell migration (9, 17). By using wild-type vs. Cav-1^−/−^ cell models, it has been demonstrated that Cav-1 presents a polarized distribution during directional migration by coordinating signalling of Src kinase and Rho-GTPase (9, 18, 19). And, last but not least, a key role for Cav-1 in the process of angiogenesis has been found (9, 20). As is evident from this brief review of the most recent literature, including Yokomori's findings, the process of VEGF-induced LSEC morphogenesis and migration and/or proliferation is controlled by different signal-transduction proteins and signalling pathways, the knowledge of which offers novel targets for the treatment of several liver sinusoidal endothelial-associated diseases – particularly, diseases where angiogenesis is central.
